# Account of Three Cases of Carditis: With Remarks

**Published:** 1834-10-01

**Authors:** William Stroud

**Affiliations:** Physician to the Northern Dispensary, &c.


*Account of Three Cases of Carditis: with Remarks.* Communicated to the Harveian Society, by William Stroud, M.D. Physician to the Northern Dispensary, &c.

The great interest and importance attached to disorders of the heart must be obvious, on the slightest consideration of the universal and powerful influence exercised by that central organ on every other part and function of the body. The difficulty of discriminating some of these disorders, more especially the inflammatory affections, has been acknowledged by the latest and best authors on the subject; since, as in other cases, prominent and alarming symptoms are often comparatively insignificant, and severe diseases may, for a while, be concealed under obscure and ambiguous indications. This difficulty of distinction, which more or less belongs to the general class of cardiac complaints, becomes still greater, as might naturally be expected, in reference to their subordinate details. For, in the heart, as in other complex organs, different parts may be separately or conjointly affected; the pericardium, the lining membrane, the muscular fibres, or the valvular apparatus: the actions of these several parts may be variously increased, diminished, or perverted; and the resulting structural derangements, whether atrophy, or hypertrophy, contraction, or dilatation, adhesion, or effusion, may be equally diversified. Among these affections, inflammation of the pericardium may, as is well known, exist independently of any other; but inflammation of the heart itself, however limited in its commencement, seldom fails, sooner or later, to become universal. The importance of an early and accurate diagnosis of these disorders is equal to its difficulty; since, like other inflammations, when promptly and powerfully counteracted, they are in a considerable degree subject to medical control; but, when neglected, or injudiciously treated, they too often produce incurable organic lesions, by which life is first embittered, and then curtailed.

The following cases are offered as a small contribution to the general stock of medical facts illustrative of this subject; and, as far as the opportunities of observation would permit, are related with clinical detail, in order that the reader may be placed, as nearly as possible, in the situation of a spectator, and enabled to judge, and reason for himself. The first was a case of obscure carditis, complicated with cephalic symptoms, which, not having been recognised in its earlier stage, presented for a while an unfavourable and threatening aspect; but, when it was at length ascertained, yielded completely, although gradually, to repeated bleeding, and other antiphlogistic treatment, carried to a greater extent than at first seemed to be admissible. The two other cases, which might probably be attributed to irregular and intemperate habits of life, are examples of similar, but more aggravated complaint; and are chiefly adduced to illustrate some of the final results of protracted cardiac inflammation, and to show how long, under certain circumstances, life may be preserved, and some of its most important functions tolerably well performed, under the pressure of so fatal a disease.

November 15th, 1831. I was called to visit, as a patient of the Northern Dispensary, Frances H... e, a married woman, thirty-one years of age, who has been four times delivered; the last time, seven weeks since, of a daughter, whom she suckles with difficulty, owing to the scantiness of her milk. She has laboured, during the last fortnight, under a febrile affection, with pain in the back of the head, and at the pit of the stomach; as also in the loins, and limbs. The skin is generally hot, but the feet are sometimes cold. She has a slight cough, sleeps little, and is occasionally delirious. The pulse at the wrist is above 130, and weak. The tongue is white, with red edges. She has some thirst, and no appetite. The bowels, which are easily relaxed, have been open to day, but were previously confined. The urine is natural. The cause of the complaint, which was at first regarded as continued fever, is not very obvious, except a severe chill, followed by fatigue, anxiety, and watching, while attending a sick child; and exposure of the chest to the night air, while giving suck. About six years since, the back of her head was injured by a blow, followed by an abscess, and much local pain.

*Prescriptions.* Temporibus affigantur hirud. viij. Aquæ puræ f℥v.; Liq. Ammon. Acet. f℥iij.; Tinct. Hyoscyam. Liq. Ant. tart. *aa* fℨii.; Spir. Æther. Nitric. fℨiij.; Sumatur f℥j. ter in dies.

18th. The leeches extracted dark-coloured blood, but the symptoms are little altered. The suction of the breast occasions pain at the pit of the stomach, but the infant seems to suffer no inconvenience.

*Prescriptions.* Inter scapulas admov. Empl. Cantharid. Hydrarg. Submur. gr. ij.; Pulv. Rhei gr. viij.; Sumantur primo mane, prout opus fuerit. Aquæ puræ f℥vi.; Potass. Nitrat. ℨj.; Pulv. Acaciæ gummi, ℨiv.; Tinct. Hyoscyam. fℨj.; Sumatur f℥j. ter in dies. Abrad. capillities; et removeatur infans a mamma.

20th. The head has been shaved, and the child is withdrawn from the breast. The blister afforded some relief, but the back of the head is still painful, with occasional delirium, disturbed sleep, and coldness of the feet. The bowels have been open without the aid of medicine. The pulse is nearly 140, weak, and indistinct. The tongue is white in the middle, with less redness of edges. She has a troublesome cough, with hurried respiration.

*Prescriptions.* Sumatur primo mane, prout opus fuerit, Ol. Ricini, fℨj. vel ij.—Mist. Camphor. f℥iv.; Liq. Ammon. Acet. f℥ij.; Pulv. Acaciæ gummi, ℨiv.; Spir. Ammon. Arom. m. xv.; Tinct. Hyoscyam. fℨiss.; Sumatur f℥j. quater in dies; et Extract. Hyoscyam. gr. v. omni vesp. horâ somni.

22d. The bowels are spontaneously open. The pain in the back of the head is severe, and is accompanied with some intolerance of light, and of sound, florid complexion, and contracted pupils. The sleep is still interrupted, and she is occasionally delirious. The cough is attended with expectoration, and, after coughing, she has several times to day vomited a mucous liquid. The tongue is now natural.

*Prescriptions.* Nucha extrahantur per cucurb. sang. f℥viij. vel xij.; Axillis inung. omni vesp. Ung. Hydrarg. Camphor. ʒj. Aquæ puræ f℥vj.; Potass. Nitrat. ʒj.; Pulv. Acaciæ gummi, ʒiij.; Tinct. Zingiber. fʒij.; Liq. Ant. tart. fʒiv. Sum. f℥j. ter, vel quater in dies. Rep. Ol. Ricin. prout opus fuerit.

23d. The pain of the back of the head was relieved by the cupping, but there still remains some intolerance of light, and of sound, with disturbed sleep, slight delirium, and noise in the ears. The mixture excites vomiting. The bowels, and urine are natural. The cough is troublesome, and is accompanied with hurried breathing, white frothy expectoration, and pain in the middle of the chest. The pulse is from 120 to 140, and extremely small and weak.

*Prescriptions.* Extrahr. per V. S. sang. f℥viij. vel xij. Pectori superiori impon. Emplastr. Cantharid. Aquæ puræ f℥vj.; Potass. Nitrat. ʒj.; Pulv. Acaciæ gummi, ʒiij.; Tinct. Hyoscyam. fʒj.; Vin. Ipecacuan. fʒij. Sum. f℥j. ter vel quater in dies. Rep. Unguent. Hyd. Camphor. et p. o. f. Ol. Ricini.

25th. The bleeding was well borne. The blood drawn yielded a coagulum covered with a thin, red coat, but was otherwise nearly natural. The blister did not produce much effect. The gums are slightly influenced by the mercurial ointment. The bowels are open. The headach and nausea are removed, but there is still a morbid sound in the ears, and the sleep is unquiet. The cough is troublesome, and the expectoration scanty. The pulse is 130, and feeble, with a sense of internal debility.

*Prescriptions.* Mist. Camph. f℥iv.; Liq. Ammon. Acet. f℥ij.; Spir. Ammon. Aromat. m. xx.; Tinct. Scillæ, fʒj.; Tinct. Hyoscyam. fʒiss. Sumr. f℥j. quater in dies.—Emplas. Canth., Ung. Cetacei, āā ʒiv.; Antim. Tart. ʒj.; Ol. Camph. fʒij. Fiat ceratum inter scapulas illinendum.—Rep. Ung. Hydrarg. Camphor. etiam p. o. f. Ol. Ricini.

27th. The antimonial ointment has produced little effect. The mixture is said to be too heating. The bowels are open. The sleep is unquiet, and attended with slight convulsions. The noise in the ears continues, but without headach or nausea. The cough is nearly dry, the breathing is hurried, and the patient is in a state of disquietude and delirium, with a disposition to uncover herself, and to get out of bed. The pulse is very frequent, and feeble. The respiratory murmur, now for the first time examined, is sufficiently audible.

*Prescriptions.* Extrahr. per V.S. sang. f℥viij. vel xij.—Pulv. Rhei, gr. iv.; Magnes. Carbonat. gr. viij. Sumr. primo mane, p. o. f.—Aquæ puræ, f℥iv.; Liq. Ammon. Acetat. f℥ij.; Pulv. Acaciæ gummi. ʒiv.; Tinct. Digital. fʒss.; Vin. Ipecacuan. fʒj.; Tinct. Hyoscyam. fʒij. Sumr. f℥j. quater in dies.—Rep. Ung. Cantharid. cum Antim. Tart.

29th. The bleeding relieved the symptoms, without inducing syncope, although a sense of debility ensued. The blood drawn was nearly natural, but the coagulum was soft. The antimonial ointment has at length produced a powerful effect. On the whole, the patient is better, and manifests less disquietude; but her sleep is still disturbed. The action of the heart, examined this day for the first time, was found to be very strong, although the pulse at the wrist was extremely small, and weak.

*Prescriptions.* Extrahr. per V.S. sang. f℥xij. vel xvj. Præcordiis affigr. hirud. vj. vel viij. Rep. Med. præter Ung. Canth. cum Ant. Tart. cujus loco substit. Ung. Cetacei.

Dec. 3d. The bleeding, and leeches gave relief. The blood drawn presented the same appearance as before. The skin of the back is much ulcerated by the antimonial ointment. The patient is considerably better, but has still a dry cough, with hurried breathing, and a neuralgic pain in the left leg, extending to the hip. The pulse is 112, and more tranquil; as is likewise the action of the heart.

*Prescriptions.* Rep. med.

Dec. 5th. The cough, and difficulty of breathing are relieved, but, in other respects, the patient is rather worse. The pulse is 120, weak, and soft; the sleep is disturbed, and there is slight delirium. The tongue, however, is clean, and moist, and the bowels, and urine are natural. The pain in the left leg follows the course of the sciatic nerve, and is somewhat relieved by friction.

*Prescriptions.* Extrahr. per V.S. sang. f℥viij. vel xij.—Aquæ puræ f℥iv.; Liquor. Ammon. Acetat. f℥ij.; Pulv. Acaciæ gum. ʒiij.; Tinct. Digital. fʒss.: Tinct. Hyoscyam., Vin. Ipecacuan. āā fʒiss. Sumr. f℥j. quater in dies.—Rep. Ung. Cetacei dorso exulcerato.

7th. The bleeding again gave relief. The blood drawn was natural. The skin of the back is not yet healed. Since yesterday, a miliary eruption, attended with itching, has appeared on the head, and neck. A similar eruption preceded her last confinement, and was accompanied with profuse sweating, even when she was sitting still. Her sleep is disturbed by the pain of the left leg, and she has slight delirium. The heart sometimes palpitates, but the breathing is tranquil. The pulse is 120, and weak.

*Prescriptions.* Linim. Sapon. comp. f℥iij.; Tinct. Opii, fʒiv.; Fiat Liniment. cruri dolenti infricandum. Rep. Med. sed augr. Tinct. Digital. ad m. xl.

14th. The skin of the back is now nearly healed; the pain of the left leg is relieved. The patient is generally better, but her head has lately been somewhat painful, and oppressed.

*Prescriptions.* Aquæ puræ, f℥vj.; Potass. Nitrat. ʒj.; Pulv. Acaciæ gummi, ℥iij.; Tinct. Digital. fʒss.; Tinct. Hyoscyam. fʒj. Sumr. f℥j. ter in dies. Repetatur Linim. opiatum.

18th. The skin of the back is not yet entirely healed; the pain of the left leg continues, and is, perhaps, of uterine origin; as it commenced about two years since, during a confinement, and was attended with pain of the uterus, and yellow discharge.

The action of the heart is still too strong, and too rapid. The pulse at the wrist, in the sitting posture, is 140, and excessively weak. There is no cough, nor disturbance of breathing, but occasional headach, and noise in the ears.

*Prescriptions.* Mist. Camphor. f℥iv.; Liq. Amm. Acet. f℥ij.; Pulv. Acaciæ gummi, ʒiij.; Tinct. Digital. fʒss. Tinct. Hyoscyam. fʒj. Sumr. f℥j. ter in dies. Repr. Linim. opiatum.

24th. The action of the heart is again increased, and attended with pain extending into the back, by which the breathing is impeded, but there is no cough. A few leeches recently applied to the præcordia extracted a little thick and very black blood. The sleep is still disturbed, with some headach, and noise in the ears. The bowels are rather confined. The urine is natural. The pain of the left leg is relieved.

*Prescriptions.* Extrahr. per V.S. sang. f℥xij. vel xvj.—Aquæ puræ, f℥vj.; Potass. Nitrat. ʒj.; Tinct. Digital. fʒss.; Tinct. Hyoscyam. fʒj.; Liq. Antimon. Tart. fʒiij. Sumr. f℥j. ter vel quater in dies.—Ol. Ricin. fʒij. vel iij. primo mane, p. o. f. Extract. Hyoscyam. gr. v. omni vesp.

28th. The bleeding gave great relief while the blood was yet flowing. The pulse, in the recumbent posture, was recently 90, and stronger. The action of the heart is tranquil, and the breathing is easy. With the exception of noise in the ears, some remaining pain in the left leg, and a sense of coldness in the loins, indicating, perhaps, the approach of menstruation, there is little complaint.

*Prescriptions.* Lumbis admovr. Empl. Canth. Repr. Med.

January 4th, 1832. The blister operated well, but did not remove the sense of coldness in the loins. Hitherto there is neither menstruation nor white discharge. The pain in the back of the head has returned, with noise in the ears, and pain in the left leg, from the ankle to the groin; the whole, probably, depending on latent menstrual disturbance. On recent examination, the uterus was found relaxed, and the vagina dilated, as if there were some danger of prolapsus. The action of the heart is tranquil. The pulse, in the recumbent posture, is 60, and placid. The bowels and urine are natural.

*Prescriptions.* Aquæ fervent. O j.; Canell. alb., Zingib. rad. āā gr. xv.; Gentian. rad., Quassiæ ligni., Aurant. cort., āā ʒss. Post horas ij. coletur Liquor. Infusi hujus amari f℥vj.; Vin. Ferri, Vin. Aloes, āā fʒiij. Sumr. f℥j. ter in dies. Repr. p. o. f. Ol. Ricini, Extr. Hyoscyam. et Liniment. Opiat.

14th. Menstruation commenced on the 8th instant, and terminated yesterday. The discharge was sufficiently copious, but rather pale. On the first day of its appearance, there was some renewal of pain in the heart, which was relieved by a few leeches applied to the præcordia. The pulse, in the evening, and in the sitting posture, was 100. There still continue some nervous symptoms; namely, a sense of coldness in the loins, noise in the ears, pain in the back of the head, and painful swelling of the left leg, from the toes to the groin.

*Prescriptions.* Inguini sinistro affigr. hirud. vj.—Infus. Sennæ, f℥vj.; Aq. Menth. vir. f℥ij.; Magnes. Sulphat. ℥ij. Sumr. f℥j. primo mane p. o. f.—Aquæ puræ, f℥vj.; Potass. Nitrat. ʒj.; Pulv. Acaciæ gummi, ʒiij.; Tinct. Digit. m. xx.; Liq. Ant. Tart. fʒiij. Sumr. f℥j. ter in dies.

Feb. 14th. The neuralgic pain of the left leg was relieved by the leeches applied to the groin, and the cephalic symptoms by a blister, which the patient recently applied, of her own accord, to the back of her neck, and which operated very powerfully. The pulse today at the wrist, in the sitting posture, and after walking, is 120, and weak.

*Prescriptions.* Aquæ puræ f℥vj.; Potass. Nitrat. ʒj.; Tinct. Digital. m. xx; Tinct. Scillæ, fʒj.; Liq. Antimon. Tart. fʒiij. Sumr. f℥j. ter in dies. Rep. p. o. f. Mist. Purg.

The patient afterwards slowly recovered, although retaining for some time a disposition to irregularity, and undue frequency in the action of the heart. She soon became again pregnant, and, towards the end of November, was safely delivered of a daughter, to whom, as to the last, she yielded a scanty supply of milk. At the beginning of February, in the following year, owing apparently to the influence of cold and wet, she had a slight return of the cardiac affection, attended with pain at the top of the head, which three months before had been accidentally injured, as also with a renewal of the former neuralgic pain in the left thigh and leg. On this occasion the complaint was, however, much less severe, and was attended with more obvious symptoms of gastro-intestinal irritation; namely, white tongue, prominent papillæ, pimples about the nostrils, thirst, bilious vomiting, moaning respiration, bowels usually confined, but easily disturbed by purgative medicine, red scanty urine, &c. Under the use of venesection, cupping at the back of the neck, followed by a blister, with aperient and antiphlogistic medicines, she speedily recovered, and subsequently continued well.

Remarks. On taking a retrospect of this case, it evidently appears to have been an obscure, but serious inflammation of the heart and brain, which, after an active continuance of two months, terminated in gradual convalescence. The complaint had already proceeded for two weeks without control, when the patient first came under my care, about the middle of November, 1831; and, owing to the ambiguous aspect of the symptoms, which were at first mistaken for those of continued fever, another week elapsed before any active depletion was employed. Its true nature having at length been ascertained, bleeding was repeated six times in the course of a month, as circumstances seemed to demand, and afforded prompt and effectual relief, both at first, and several times afterwards, when the disorder, after a temporary check, was aggravated, or renewed. Independently of the application of a few leeches to the temples and præcordia, the whole quantity of blood drawn was, probably, between sixty and seventy ounces, of which about forty ounces were abstracted during the last week of November, and the remainder in the course of the following month. The inflammatory character of the complaint was strongly illustrated by this result, and by the benefit derived from the internal use of nitre, tartarised antimony, hyoscyamus, and digitalis, although some of these remedies seemed ill adapted to a state of apparent debility; as likewise from the counter-irritation of blisters, and more particularly of antimonial ointment, extensively applied between the shoulders, which at length produced a very powerful and durable effect, contributing, no doubt, to secure the ground already gained, and to diminish the necessity for further bleeding.

By the end of December the disease was decidedly subdued, although slight symptoms occasionally recurred for some time later. Menstruation took place in the beginning of January, and the patient was ultimately restored to perfect health.

In this interesting case, the conjoined inflammations of the heart and brain, although mutually modified, were so equal, and independent, that it was difficult to ascribe priority to either of them. In a manner strictly parallel, they began, continued, and ended together; and the same complex character was again displayed, when, from similar causes, the complaint was renewed in a milder degree a year afterwards. The principal cerebral symptoms were pain and heat of the occiput, with coldness of the feet, flushed countenance, contracted pupils, intolerance of light and of sound, noise in the ears, delirium, disquietude, and watchfulness, or imperfect sleep, disturbed by convulsive movements. The principal cardiac symptoms were pain in the præcordia and epigastrium, sometimes piercing through to the back, occasional palpitation, vomiting after coughing, violent action of the heart, discovered by auscultation, together with an extremely weak and frequent pulse at the wrist, general debility, and tendency to fainting, on making the slightest exertion.

The internal constitution of complex disorders is often analysed, and demonstrated by the variable duration and succession of their several symptoms; and thus, in the present case, the early subsidence of the remaining complaints, in proportion as those above described were relieved, proved them to be secondary affections, of which the gastric, and febrile, probably depended on the cerebral inflammation, and the pulmonary on that of the heart. The miliary eruption about the face and neck, which attended the mitigation of the disorder, like the profuse local sweat by which it was accompanied on a former occasion, a little before the patient's last confinement, might be attributed to the joint influence of both. The neuralgic pain of the left leg, partly arising from a local disposition of uterine origin, but probably renewed by the excitement of the brain, and therefore aggravated by menstrual disturbance, seems at last to have become nearly independent, maintained, apparently, by an inflammatory state of the principal nerves of the limb, and hence relieved by the application of leeches to the groin. In like manner, after pertussis, various local nervous affections are sometimes observed to survive the temporary congestion of brain, from which they originated. On the other hand, the continuance of the more simple functional derangements, such as weak and frequent pulse, slight headach, with noise in the ears, &c., during the commencement of convalescence, might reasonably be ascribed to a merely negative state of debility, remaining after the principal morbid action had ceased. The occurrence of the menstrual discharge, for the first time after delivery, was both a sign and a cause of amendment; inasmuch as that discharge, which mainly depends on a peculiar state of cerebral excitement, having been prevented by the more powerful excitement of the disease, during its active stage, served at once to attest and to accelerate its decline. The reduction of the pulse under this agency, within a few days, from 140 to 60, furnishes a striking illustration of the influence of the brain on the heart.

The predisposition to this severe attack, consisting in an irritable state of the organs principally concerned, seems to have been derived from various ordinary sources: the confinement a few weeks before; a scanty secretion of milk; domestic troubles and vexations; fatigue, anxiety, and want of sleep, while nursing a sick child; and a severe injury of the back of the head, which took place six years before, and produced at the time an abscess, and much pain. The influence of mental disturbance in generating disease is well known, but that of local injuries, from blows and falls, is not always duly appreciated, although many proofs might be adduced that it often continues to operate for several years; and, after a long and indefinite interval, may occasion very serious results. The exciting cause was probably cold, which is quite capable of inducing inflammation, both of the brain and heart, especially when thus predisposed. The whole body had been previously subjected to cold and wet; and the chest had been repeatedly chilled by imprudent exposure, while suckling an infant, during the night. The intercourse between this portion of the cutaneous surface and the contained viscera, which is at all times considerable, seems to be increased in females, during the process of lactation, and was well illustrated by the pain which, at the commencement of the case, the sucking of the child excited in the epigastrium.

Owing to the operation of the brain and the heart on each other, and on the whole body, the symptoms of this complaint were numerous, complicated, and obscure, closely resembling those of continued fever, which, indeed, there is every reason to infer, is essentially a disorder of the nervous centre, including the brain, the spinal chord, and the ganglionic system. The low delirium, the tendency to fainting on the slightest exertion, the extreme frequency and feebleness of the pulse, and other signs of apparent debility, favoured this view, and seemed totally to forbid the practice of depletion. Yet the abstraction of not less than sixty or seventy ounces of blood was afterwards found necessary; and by this, and other antiphlogistic remedies, the complaint, which had been gradually increasing, and presented a very formidable aspect, was immediately checked, and ultimately subdued. The diagnosis was, at length, determined by stethoscopic examination, which, owing to hurry and preconception, had been improperly omitted in the first instance. Other considerations might, perhaps, have led to the same conclusion, but the violent action of the heart, thus detected, and its manifest influence in compressing and irritating both the lungs and the stomach, producing cough, dyspnœa, nausea, and vomiting, afforded the clearest proof of its inflammatory condition, and the surest indication of the mode of treatment to be adopted. This furnishes another example of the value and importance of the acoustic signs of disease, which are, in general, so decisive, and so easily attainable, that they ought never to be neglected. Had they been consulted, and followed, at the commencement of the case, there is every reason to believe that it might have been promptly subdued, with far less risk, and at a much smaller expense of time and strength, than actually occurred.

Under inflammation of the pericardium, or of its contents, the pulse of the heart is not always alike, but differs with the circumstances. It is sometimes, for example, short, irritable, and energetic; sometimes soft, obscure, and indistinct; according as the muscular function of the organ is variously modified, or directed. The pulse may be either strong in the heart, and weak in the arteries, or precisely the reverse: its shock, or impulse, also, is sometimes directed to the epigastrium, sometimes to the sides of the chest, and, in some instances, seems to be more operative on the contained blood, than on the surrounding parts. The diagnosis between carditis and pericarditis is by no means easy; but the character of the latter is, probably, influenced by the serous effusion into its cavity with which, at an early period, it is usually complicated. The frequency and feebleness of the arterial pulse, in conjunction with powerful action of the heart, is an interesting and remarkable circumstance, for which, in the absence of any permanent mechanical obstruction, it is difficult to account. It may be ascribed, either to the temporary dilatation of the cardiac chambers, by which their contraction, although strong, is rendered less effective in expelling their contents; or to spasmodic constriction of the muscular fibres near the orifices of the great arteries; or to certain obscure conditions, not hitherto fully explored, in the vessels themselves, by which the circulation through them is either promoted or resisted. However produced, this condition serves to explain the mixed and ambiguous character of the symptoms in such complaints, combining excessive action with apparent weakness, and implies, perhaps, that inflammation is more dependent on local excitement than on the injecting force of the heart. It, also, serves to explain the unusual quality of the blood, which, in so inflammatory a disease, exhibited no corresponding character; the coagulum having been, for the most part, soft and red, without any tendency to separate a buffy coat; showing how much the condition of the blood depends on the vital action of the heart, and vessels, on which, in its turn, it exerts an important influence.

[The remaining cases of Carditis will be inserted in the ensuing Number.—EDITOR.]

